# Comparing surface immune markers in successful and non-viable ART pregnancies on the day of hCG measurement: a prospective pilot study

**DOI:** 10.1530/RAF-24-0034

**Published:** 2025-01-11

**Authors:** Kevin Marron, Conor Harrity

**Affiliations:** ^1^ Enfer Medical, M7 Business Park, Naas, Kildare, Ireland; ^2^ RCSI University of Medicine and Health Sciences, Dublin, Ireland; ^3^ Beaumont Hospital, Dublin, Ireland

**Keywords:** pregnancy, peripheral blood, immunophenotype, ART, lymphocytes

## Abstract

**Abstract:**

Blood lymphocyte reference ranges in non-pregnant females are established, but changes in pregnancy are less well understood. The early identification of immunological markers that could suggest an increased risk of early pregnancy loss may allow for timely intervention to improve outcomes. A lymphocytic immunophenotype provides a broad assessment of important immune parameters and potential indicators, which may be of relevance to pregnancy outcome. Comparison of immunophenotype results on the day of a positive hCG after embryo transfer between successful and failed pregnancies allows for this assessment. Baseline non-pregnant lymphocyte percentage and cell/µL profiles were established with a comprehensive panel on 93 age-matched male factor controls. Sixty-five *in-vitro *fertilisation (IVF) patients had an immunophenotype assessment on the day of a positive hCG, followed by further hCG tests and ultrasound monitoring as required to ultimately evaluate success (live birth) or failure (miscarriage). Thirty-one pregnancies were viable, leading to a live birth, while 34 ended in miscarriage. Total CD56, pNK, NKT, CD4 and CD8 levels were equivalent between all groups. Regardless of the outcome, B lymphocytes increased in pregnancy compared to controls. Of interest, in the later miscarriage cohort, pNK-specific CD69 was reduced (1.6 vs 5.4%, *P* = 0.02), while CD57+ cells were increased (45.4 vs 38.9%, *P* = 0.025). Corresponding changes were observed in cell/µL concentrations. Low level CD69 activation and elevated CD56^dim^ and CD57+ NK cells were identified as markers that could potentially identify a pregnancy at risk of miscarriage, with further study needed to explore whether these changes represent cause or effect.

**Lay summary:**

Unexplained infertility remains a difficult issue for patients and physicians alike, but despite recent diagnostic strides and innovative methods, there are no clear solutions on the horizon. Pregnancies can still occur in these challenging populations, either spontaneously or by interventions such as IVF. The early identification of various immune markers by blood sampling that may correlate with the subsequent outcome could be beneficial in identifying pregnancies at increased risk of miscarriage and perhaps allowing for timely and effective interventions.

## Introduction

Analytical and experimental epidemiological observations spanning decades have shown many finely tuned maternal modifications in the cellular and molecular immune response to pregnancy. These responses are physiologically demonstrated in a variety of forms, most notably by inhibition of allograft rejection, suppression of autoimmune disease and increased risk of maternal infection (Kraus *et al.* 2012, Pazos *et al.* 2012, Aghaeepour *et al.* 2017). In preparation for pregnancy, maternal innate and adaptive local immune responses at the trophoblast interface are fundamentally altered to make way for the fetal allograft and prevent its summary rejection (Davies 2007). Numerous key plasma proteomes involved in embryogenesis, angiogenesis, immunoregulation and inflammation are known to dramatically change expression profiles across the pregnancy and postpartum period (Romero *et al.* 2017). These critical adaptations protect the mother from harmful pathogens while simultaneously allowing for tolerance of fetal antigens (Mor *et al.* 2011).

Natural killer (NK) cell facilitation at the trophoblast–embryo interface and some top-level changes, such as systemic interleukin (IL)-4 levels and Th2 T cell modifications, are well established (Lin *et al.* 1993, Saito *et al.* 1999, Kwak-Kim & Gilman-Sachs 2008). Many factors are certainly in play all at once. Immune-specific markers of either a successful or failing early pregnancy, however, have not yet been identified (Parham 2004, Lash & Bulmer 2011). The majority of failing pregnancies in aged or IVF patients are considered to be genetic (Suzumori & Sugiura-Ogasawara 2010) or thrombotic (Rai 2003) in nature; however, it is possible that (peripheral blood) immune cell alterations may be influential and detectable at an early stage, but to what extent is largely unknown. We set out to address this issue in this study, albeit in IVF patients not necessarily representative of the general population.

The maternal immune priorities in advance of pregnancy may well be a factor in conditions such as infertility, implantation failure and poor placentation and further downstream complications such as pre-eclampsia (Moffett & Hiby 2007, Sargent *et al.* 2007, Kwak-Kim & Gilman-Sachs 2008). Recurrent pregnancy loss remains unexplained in 40–50% of cases (Stephenson 1996, Ford & Schust 2009, Hyde & Schust 2015). Normally involved in the maintenance of pregnancy, immune dysregulation is becoming more accepted as a causative mechanism behind many failed pregnancies, and possibly infertility. Most immunological investigations have focused on the natural killer cell and, more recently, NK cells in the uterine environment (Marron & Harrity 2019). The importance of dysfunctional NK cells in the aetiology of recurrent pregnancy loss is still unclear. Tregs and their contribution to successful pregnancies have been well studied, but mostly before, rather than after, implantation (Schlossberger *et al.* 2013). Treg’s influence is clearly fundamental and is closely associated with the classic ‘Th2 shift’ to a more anti-inflammatory phenotype. This switch, however, only takes place many weeks into a well-established first trimester pregnancy (Sykes *et al.* 2012, Ruocco *et al.* 2014, Feyaerts *et al.* 2017), and low Treg levels have previously been associated with spontaneous abortion (Lee *et al.* 2012). Classical T cells, in contrast, primarily provide cell-mediated immunity (Abrams & Miller 2011). T helper cells (CD4+) can be further subdivided into T helper 1 (Th1) cells and T helper 2 (Th2) cells depending on their pattern of cytokine production. Th1 cells secrete pro-inflammatory cytokines, such as interferon gamma (IFN-γ) and tumour necrosis factor alpha (TNF-α), whereas the Th2 cells secrete anti-inflammatory cytokines, such as IL-4, IL-10 and IL-13.

Using a comprehensive flow panel, we describe the peripheral blood NK variants, CD4+ T cells and subtypes, CD8+ T cells and B lymphocytes in non-pregnant age-matched controls and early pregnancy, in an attempt to identify a lymphocytic subtype that may be an indicator of pregnancies that later lead to a live birth vs those that suffer pregnancy loss. An improved understanding of the immune mechanisms involved in implantation and early pregnancy establishment could pave the way for treatment modifications to optimize diagnosis, prognosis and care, thereby improving fetal growth, development and live birth (Loke & King 2000, Kraus *et al.* 2012).

## Subjects and methods

To establish the non-pregnant baseline immune pattern for the surface lymphocytic markers under investigation, 93 patients from a university-affiliated ART centre had a peripheral blood reproductive immunophenotype performed for analysis. This control group was selected from couples with primary male factor aetiology, where all female diagnostic investigations were normal, with no known history of endometriosis or other medical disorders and normal ultrasound imaging, in order to approach as closely to a normal fertile female population immunophenotype as possible. A peripheral blood immunophenotype in the control group was not performed on a specified day of the menstrual cycle but was performed in the early follicular phase at their baseline assessment, pre-treatment, between cycle days 2 and 5 to maintain consistency. An additional 65 test samples were obtained from IVF patients with unexplained infertility taken alongside a positive serum hCG test 14 days following frozen embryo transfer. Medical history was reviewed for these patients to ensure the aetiology was unexplained and to exclude potential confounding factors for miscarriage, with none having identifiable sperm abnormalities, or female risk factors, such as anatomical disorders. Unfortunately, pre-implantation genetic testing-aneuploidy (PGT-a) was not incorporated into the treatment cycles to exclude the transfer of aneuploid embryos. Only medicated frozen embryo transfer cycles were included to ensure consistency and exclude heterogeneity associated with stimulated cycles (e.g. high oestrogen levels, number of follicles and potential alterations to endometrial receptivity).

The testing laboratory has extensive experience with immunophenotyping by flow cytometry for peripheral and menstrual blood and endometrial biopsies (Marron *et al.* 2019, Marron & Harrity 2022). While different to the control group, who have an identifiable male factor aetiology, the ART study group have comparable baseline characteristics. The patient populations studied may not necessarily represent those of the general reproductive age population, and given their clinical history, pregnancy loss in these particular individuals may certainly be multifactorial rather than being specifically immune mediated. Due to financial constraints, the design of this pilot study did not allow for prior baseline immune evaluation in the study population, or chromosomal assessment of the embryos prior to transfer.

Peripheral blood samples in EDTA were procured by venepuncture and analysed by flow cytometry within 24 h of an automated immunoassay-based pregnancy test to establish serum βhCG levels. Standard peripheral blood, no-wash and lyse FCM protocols to minimise cell loss were utilised. Briefly, 100 μL peripheral blood were added to a cocktail of 100 μL antibodies + staining buffer as described below and incubated at room temperature for 20 min. Nine hundred microlitres Versalyse™ solution (Beckman Coulter UK Ltd) were used to eliminate red blood cell contamination prior to analysis on Navios 10C FCM (Beckman Coulter, UK), while the final 100 μL volume were contributed by Flow Count™ (Beckman Coulter, UK) beads. These beads allow concentration estimates to be calculated automatically in cells per µL of the sample.

Co-localisation of selected antibodies was employed across individual tubes for cellular evaluation, with a 10 colour flow panel and appropriate compensation matrices as described above (Marron *et al.* 2018); see Supplementary Table (see section on Supplementary materials given at the end of the article) for details. Cell types were defined according to accepted conventions. Tube 1 assessed peripheral type NK (pNK; CD16+ and CD56^dim^) cells, natural killer T (NKT; CD3+, CD16− and CD56^dim^) cells, expression of CD69 activation marker and CD57 NK maturity marker (within pNK and NKT), naive B lymphocytes (CD19+) and B1 type lymphocytes (CD5+ and CD19+). Tube 2 allowed analysis of T lymphocytes (CD4+ and CD8+) and various CD4 subsets, including Th1 (CD4+, CD183+ and CD196−), Th2 (CD4+, CD183− and CD196−) and regulatory T cells (Treg; CD4+, CD127^dim^ and CD25^bright^). Splitting the tubes allows more markers to be assessed using the same fluorophores without fear of crossover cell contamination. Both percentages and cell counts per µL were assessed. 7-AAD cell viability assessments showed immune cell apoptosis to be minimal within the time frame of the study performance.

Informed consent was obtained from all patients for the analysis, with advanced approval obtained from the clinic’s research ethics committee and quality management department. The non-parametric Kruskal–Wallis rank test was used to look for *P* value differences (<0.05) between the control, viable and miscarriage cohorts. The Kruskal–Wallis test was selected for comparing the overall differences between multiple groups as it is an omnibus test, controlling for an overall false-positive rate and thus reducing the risk of type 1 errors. For direct comparison between two groups, a further assessment with the Wilcoxon rank-sum test was performed. IBM SPSS v24 was employed for statistical analysis.

## Results

Using flow cytometric side scatter and CD45+ gating, baseline immunophenotype lymphocyte percentages and concentrations (cells/µL) were established in 93 non-pregnant age-matched individuals, with male factor infertility, creating a control group (Table 1). Further immunophenotyping techniques with antibody cocktails and specific laser wavelengths allowed lymphocytes to be clearly differentiated from the other mononuclear leucocytic cells present and demonstrated that peripheral blood possesses diverse lymphocyte subpopulations comprising T cells, B cells, peripheral type natural killer (pNK) cells and natural killer T cells (NKT) cells. Distinct monocyte and polymorphonuclear cell populations were also present but were not characterised in this study. Of the 65 positive hCG tests post-embryo transfer, 31 were later found to be viable and lead to a live birth, while 34 ended in miscarriage, dividing the initially pregnant population into two cohorts. Demographic differences between the groups are illustrated in Table 1 and highlight the differences between the pregnant and control populations in terms of reproductive history.

**Table 1:** Demographic details found between the study population groups.

	Controls	Viable	Miscarriage
*n*	93	31	34
Age	36.8	37.8	36.3
Sample timing	Baseline (CD 2–5)	D14 after ET	D14 after ET
hCG range	N/A	144–1772	20–1340
Prior LB	0	9	8
Previous miscarriage	0	12	14

CD, cycle day; ET, embryo transfer.

**Figure 1:**
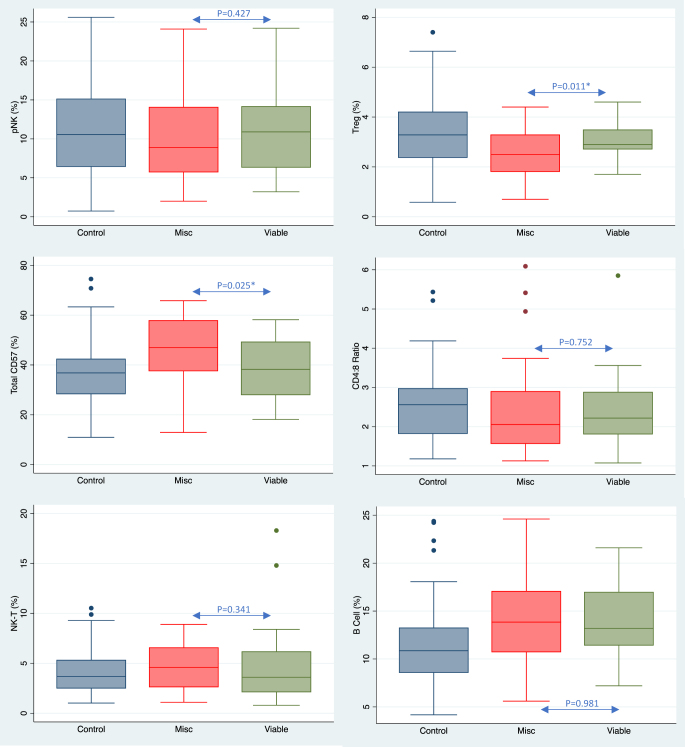
Box plot demonstrating the distribution of lymphocyte values between non-pregnant controls, and both miscarriage and viable pregnancy groups.

Comparing results between the control and the overall combined pregnancy group, it was shown that percentage proportions and concentrations of CD56+ cells (pNK and NKT) and both CD4+ and CD8+ T lymphocytes were the same between all cohorts (Tables 2 and 3), with no identified changes relative to controls. B cell (CD19+) lymphocytes were significantly elevated relative to controls in both proportions (14.1 vs 11.5%, *P* = 0.0001) and concentrations (348.9 vs 255.7 cells/µL, *P* = 0.0001), while pNK CD57 showed elevated levels in these patients (Fig. 1). Unexpectedly, the early pregnancy cases had lower concentrations of Tregs and NK CD69+ activation relative to the male-factor controls (Tables 2 and 3).

**Table 2:** Mean lymphocyte proportions (%) and concentrations (#; cells/μL) with associated standard deviation (SD) between the non-pregnant control group (normal female population with male-factor aetiology) and immunophenotype on the day of a positive hCG. The Wilcoxon rank-sum test was used to compare differences between groups.

Cell type	Control (%) (*n* = 93)	hCG+ve (%) (*n* = 65)	*P* values	Control (#) (*n* = 93)	hCG+ve (#) (*n* = 65)	*P* values
*pNK*	11.2 (*5.5*)	10.6 (*5.6*)	0.36	241.0 (*133.9*)	233.3(*144.9*)	0.51
*CD69*	5.1 (*7.0*)	3.4 (*6.9*)	0.016*	17.1 (*27.1*)	10.4 (*17.1*)	0.030*
*NKT*	4.3 (*2.1*)	4.7 (*3.2*)	0.81	91.3 (*53.5*)	109.9 (*80.8*)	0.30
*CD56*	15.8 (*6.2*)	15.9 (*6.7*)	0.93	335.2 (*159.8*)	353.9 (*178.2*)	0.53
***CD57***	37.0 (*12.6*)	42.3 (*12.9*)	0.007*	129.7 (*89.4*)	160.0 (*98.5*)	0.031*
*CD4*	45.6 (*7.0*)	43.5 (*8.5*)	0.072	977.6(*417.4*)	991.4 (*481.9*)	0.96
*CD8*	19.5 (*5.3*)	20.0 (*6.1*)	0.56	414.4 (*180.3*)	452.8 (*210.8*)	0.29
*CD4:CD8*	2.5 (*0.9*)	2.9 (*4.2*)	0.14	-	-	-
*Treg*	3.3 (*1.4*)	2.5 (*1.1*)	0.0004*	31.7 (*18.4*)	23.2 (*12.4*)	0.006*
*Th1*	22.8 (*8.9*)	22.2 (*9.4*)	0.37	212.5 (*111.2*)	207.4 (*102.7*)	0.89
*Th2*	52.23(*10.7*)	55.2 (*11.2*)	0.22	516.0 (*241.8*)	556.1 (*321.2*)	0.75
*Th1:Th2*	0.75 (*2.8*)	0.43 (*0.2*)	0.41	-	-	-
*B cell*	11.5 (*3.9*)	14.1 (*4.2*)	0.0001*	255.7 (*146.9*)	348.9 (*229.8*)	0.0001*

*bold font indicates statistical significance.

**Table 3:** Mean lymphocyte proportions (%) and concentrations (#; cell/µL) with associated standard deviation (SD) between pregnancies that continued on as viable or finished with miscarriage, measured on the day of a positive hCG. The Kruskal–Wallis rank test was used to compare differences between groups.

Cell type	Viable (%) (*n* = 31)	Misc (%) (*n* = 34)	*P* values	Viable (#) (*n* = 31)	Misc (#) (*n* = 34)	*P* values
*pNK*	11.0 (*5.1*)	10.2 (*6.0*)	0.43	227.2 (*144.1*)	239.8 (*144.1*)	0.83
***CD69***	5.4 (*1.7*)	1.6 (*1.3*)	0.02*	5.7 (*5.8*)	15.7 (*5.8*)	*0.062*
*NKT*	4.6 (*3.8*)	4.7 (*2.5*)	0.34	118.3 (*74.1*)	100.9 (*74.1*)	0.15
*CD56*	16.1 (*6.6*)	15.7 (*6.9*)	0.80	357.6 (*175.0*)	349.8 (*175.0*)	0.74
***CD57***	38.9 (*11.8*)	45.4 (*13.3*)	0.03*	172.1 (*101.6*)	146.7 (*101.6*)	*0.28*
*CD4*	42.4 (*8.2*)	44.6 (*8.7*)	0.53	1015.2 (*395.9*)	965.3 (*395.9*)	0.31
*CD8*	19.1 (*5.4*)	20.8 (*6.5*)	0.46	471.4 (*206.2*)	432.5 (*206.2*)	0.53
*CD4:CD8*	3.4 (*6.0*)	2.5 (*1.2*)	0.75	-	-	-
*Treg*	2.5 (*1.2*)	2.5 (*1.0*)	0.76	24.6 (*12.4*)	21.7 (*12.4*)	*0.33*
*Th1*	22.8 (*9.0*)	22.2 (*9.4*)	0.51	215.4 (*107.4*)	198.7 (*107.4*)	0.63
*Th2*	54.8 (*11.7*)	55.5 (*11.0*)	0.94	570.7 (*278.8*)	540.1 (*278.8*)	0.35
*Th1:Th2*	0.5 (*0.3*)	0.4 (*0.2*)	0.77	-	-	-
*B cell*	14.1 (*3.9*)	14.1	0.98	357.1 (*233.6*)	339.8 (*233.6*)	0.68

*bold font indicates significant markers and their associated *P* values.

**Figure 2:**
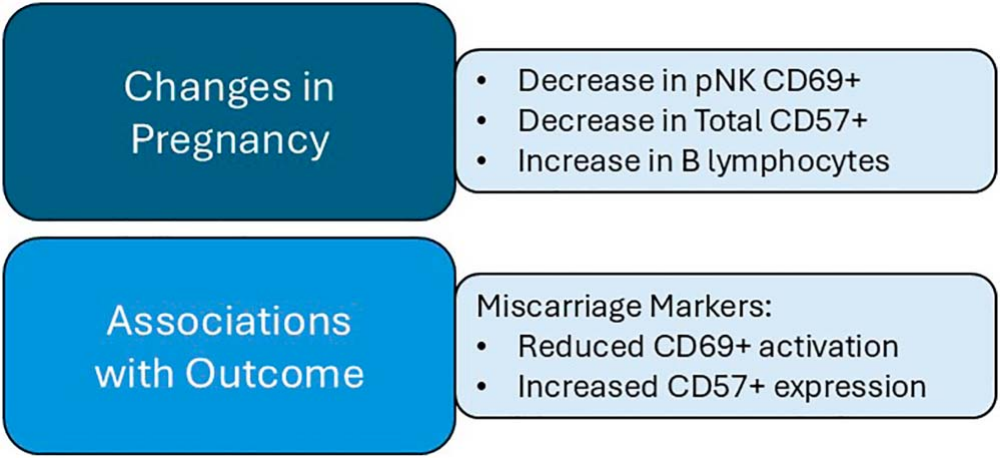
Summary of the immune changes seen in response to or as a cause of failing or viable pregnancies.

When the pregnant population was subdivided into ongoing viable vs non-viable groups, several significant differences became apparent. The peripheral blood natural killer (pNK) cell CD69+ activation was markedly reduced in the miscarriage cohort, in both percentage expressions (1.6 vs 5.4%, *P* = 0.024) and concentrations (5.7 vs 15.7 cells/µL, Table 3) and an increase in CD57+ NK cells (comprising both CD16+CD56^dim^ pNK and CD3+CD56^dim^ NKT) (45.4 vs 38.9%, *P* = 0.025) (Fig. 2). Within the pregnancy study groups, Treg levels showed equivalence when tested at cycle day 15. Understanding the mechanisms for these changes requires additional investigation.

## Discussion

Normal pre-pregnancy patterns of peripheral blood lymphocyte subtypes have been established, but the impact of immune dysregulation on reproductive function is poorly understood. Immunological patterns and changes with pregnancy are both extensive and obscure. Apart from the male-factor control samples, the study cases had a poor reproductive history, with repeated episodes of pregnancy loss or implantation failure of unexplained aetiology on a background of prolonged infertility, so the changes observed may not exactly mirror those in a more ‘normal’ pregnant population. Other design flaws are present in this pilot program. Unfortunately, the study group does not benefit from a baseline pre-treatment or non-pregnant immunophenotype, so they are compared with controls where the sample was collected in the follicular phase. Some studies have suggested that the immunophenotype may vary over a menstrual cycle (Oertelt-Prigione 2012). This could be influenced by progesterone having immunosuppressive type properties, favouring a Th2 cytokine profile and inhibiting mast cell degranulation (Zwahlen & Stute 2024). Further comparison of follicular and luteal phase reproductive immunophenotypes would help clarify this potential discrepancy.

The importance of classical T regulatory cells in the establishment of pregnancy rivals that of natural killer cell involvement in the process. The immunosuppressive properties of these cells are important in developing maternal tolerance to fetal alloantigens and establishing a viable pregnancy (Huang *et al.* 2020). Indeed, insufficient Treg numbers or function has been associated with infertility, miscarriage and pregnancy complications (Robertson *et al.* 2018). The expansion and recruitment of Tregs is hormonally sensitive, stimulated by oestrogen, progesterone and human chorionic gonadotropin (Huang *et al.* 2020). An unexpected finding in this study was higher Treg populations in the control subjects than in the early pregnancy cases and no change in Treg status between viable and failing pregnancies, at least at this very early time point. Reasons for this are difficult to elucidate, and further study is needed to determine whether this is a genuine finding, or a result of patient characteristics in the reproductive failure group and/or sample timing in the controls. Potentially, the unexplained infertility population may have lower baseline Treg levels when compared to a normal female population. As Tregs are inducible, initial low levels may have been overcome as the viable pregnancies progressed. A previous study suggested that Tregs may be preferentially recruited to the decidua of failing pregnancies (Sasaki *et al.* 2004). In addition, insufficient expansion of regulatory T cells towards paternal antigens is another proposed mechanism of pregnancy failure (Zenclussen *et al.* 2005). Treg cells are also said to be involved in the classical Th2 shift towards humoural-based immunity, as a consequence of normal pregnancy, but is not expected at this very early stage of gestation. Treg decreases in pregnancy have been described before but primarily in the identification of patients who may be pre-disposed to the development of pre-eclampsia (Salazar Garcia *et al.* 2018). In this assessment of immunophenotype changes in very early pregnancy, there was no observable change in Treg proportion or concentration when viable and miscarriage groups were compared.

The B cell changes observed here, where values are increased in both viable vs miscarriage cohorts, relative to non-pregnant controls, seem not to be useful in determining later viability. B cell changes are certainly important in the maintenance of pregnancy humoural equilibrium but again may not be manifest at the early stage studied here.

Natural killer cells have been extensively studied in the context of reproductive failure, with high proportions and increased cytotoxicity reported as poor prognostic indicators (Kwak *et al.* 1995, Dosiou & Giudice 2005). This does not preclude their intimate involvement in decidualisation, tolerisation and spiral artery remodelling and thereby successful pregnancies, but then, dysfunction may be linked to reproductive failure. CD56^dim^CD69+ pNK cells represent those cells that are positively activated over and above the baseline levels of activation. A decreased level of CD69 activation in the patients in the miscarriage cohort may therefore represent dysfunction in this important cell type. Similarly, the hyperpotent cytotoxic marker CD56^dim^CD57+ was elevated relative to controls, perhaps indicating the baseline level of this marker was raised in our study subjects. The elevated CD57 seen in the miscarriage group may simply reflect higher baseline levels of this marker, prior to pregnancy establishment, and may herald those cycles destined for immune mediated miscarriage.

A direct immune assessment of the endometrium by biopsy has been proposed as the best test to identify specific patients with poor reproductive outcomes who may benefit from targeted immunotherapy (Sacks 2015). Our previous work has identified that three immune cell surface markers (Th1 percentage, NKT and total CD57+) have a strong correlation in expression between both peripheral blood and endometrium (Marron & Harrity 2023). Although it is well established that no relationship exists between pNK cells in both compartments, there is a hypothesis that perhaps some important endometrial markers may have a direct correlation with a peripheral blood immunophenotype, perhaps reducing the need for an invasive biopsy in certain patients. The study findings here again highlight the potential significance of specifically elevated CD57+ NK cells as a marker for increased risk of pregnancy loss.

Early identification of immunological markers that could indicate an increased risk of pregnancy loss could allow for timely intervention in an attempt to improve outcome. Low NK CD69 activation and elevated CD56^dim^CD57+ NK cells have all been identified in this study as accessible surface markers that could potentially identify a pregnancy at increased risk. Whether these changes are a response to an already failing pregnancy, perhaps of chromosomal aetiology, or an immune-mediated cause or risk factor for pregnancy loss needs further study.

In conclusion, peripheral blood lymphocyte examination may be meritorious in identifying patient subgroups in which immune system dysfunction may be a factor in adverse reproductive outcomes, potentially for the maintenance of pregnancy and risk of miscarriage. Further research is needed to confirm whether the identified changes in these markers correlate with adverse outcomes in a larger more diverse population and whether the variations in identified markers are a response to a pregnancy already in the process of failing or are instead a marker of immune dysfunction that may lead to an alloimmune cause of miscarriage. Once a relationship between cause and effect has been confirmed, personalised interventional studies on selected patients could be considered to determine whether specific immunomodulatory treatments, tuned to the individual immunophenotype exhibited, could have a role in reducing the miscarriage potential for high-risk pregnancies, and possibly establishing a pathway back to viability.

## Supplementary materials



## Declaration of interest

The authors declare that there is no conflict of interest that could be perceived as prejudicing the impartiality of the work. C Harrity is an Associate Editor of *Reproduction *&* Fertility* and was not involved in the review or editorial process for this paper, on which he is listed as an author.

## Funding

This pilot study was funded locally.

## Author contribution statement

 KM conceived the projected and performed the flow cytometry analysis and CH assisted with study design, statistical analysis and editing the manuscript.

## Patient consent and ethics approval

Informed consent was obtained from all individual participants included in the study, and the protocol was approved by the clinic’s research ethics committee.
