# Supplementary material for: Comparing surface immune markers in successful and non-viable ART pregnancies on the day of hCG measurement: a prospective pilot study
Source: Reprod Fertil. 2025 Jan 11;6(1):e240034. doi: 10.1530/RAF-24-0034 (PMC11968024; doi:10.1530/RAF-24-0034)
Supplement: Supplementary file 1 [file supplementary_materials.pdf]

Laser	405 Excitation		488 Excitation					633 Excitation			
Fluorophore	Pacific Blue	Krome Orange	FITC	PE	ECD	PC5.5	PC7	APC	APC-AF700	APC-AF750	**Tube 1**
CD marker	**CD3**	**CD45**	**CD57**	**CD56**	**CD16**	**CD19**		**CD69**	**CD5**		
Clone	UCHT1	J.33	NC1	N901	3G8	J3-119		TP7.55.3	BL1a		

	405 Excitation		488 Excitation					633 Excitation			
	Pacific Blue	Krome Orange	FITC	PE	ECD	PC5.5	PC7	APC	APC-AF700	APC-AF750	**Tube 2**
	**CD3**	**CD45**	**CD127**	**CXCR3 (CD183)**	**CD8**		**CCR6 (CD196)**	**CD25**		**CD4**	
	UCHT1	J.33	R34.34	GO25H7	SFC121		B-R35	B1.49.9		13B8.2	

Supplementary Table.

Fluorphores used for flow cytometry (Navios ™ Flow Cytometer, Beckman Coulter)
